# Impact of an Interdisciplinary Deep Brain Stimulation Screening Model on Post-Surgical Complications in Essential Tremor Patients

**DOI:** 10.1371/journal.pone.0145623

**Published:** 2015-12-28

**Authors:** Masa-aki Higuchi, Dan D. Topiol, Bilal Ahmed, Hokuto Morita, Samuel Carbunaru, Christopher W. Hess, Dawn Bowers, Herbert E. Ward, Lisa R. Warren, Meredith M. DeFranco, Michelle S. Troche, Shankar J. Kulkarni, Erin Hastings, Kelly D. Foote, Michael S. Okun, Daniel Martinez-Ramirez

**Affiliations:** 1 Department of Neurology, University of Florida College of Medicine, Center for Movement Disorders and Neurorestoration, Gainesville, Florida, United States of America; 2 Department of Neurosurgery, University of Florida College of Medicine, Gainesville, Florida, United States of America; 3 Department of Psychiatry, University of Florida College of Medicine, Gainesville, Florida, United States of America; 4 Department of Clinical and Health Psychology, University of Florida College of Public Health and Health Professions, Gainesville, Florida, United States of America; 5 Department of Speech, Language, and Hearing Sciences, University of Florida College of Public Health and Health Professions, Gainesville, Florida, United States of America; 6 Rehabilitation Services, University of Florida Center for Movement Disorders and Neurorestoration, Gainesville, Florida, United States of America; University of Pennsylvania Perelman School of Medicine, UNITED STATES

## Abstract

**Objective:**

To investigate the relationship of our interdisciplinary screening process on post-operative unintended hospitalizations and quality of life.

**Background:**

There are currently no standardized criteria for selection of appropriate Deep Brain Stimulation candidates and little hard data exists to support the use of any singular method.

**Methods:**

An Essential Tremor cohort was selected from our institutional Deep Brain Stimulation database. The interdisciplinary model utilized seven specialties who pre-operatively screened all potential Deep Brain Stimulation candidates. Concerns for surgery raised by each specialty were documented and classified as none, minor, or major. Charts were reviewed to identify unintended hospitalizations and quality of life measurements at 1 year post-surgery.

**Results:**

Eighty-six percent (44/51) of the potential screened candidates were approved for Deep Brain Stimulation. Eight (18%) patients had an unintended hospitalization during the follow-up period. Patients with minor or major concerns raised by any specialty service had significantly more unintended hospitalizations when compared to patients without concerns (75% vs. 25%, p < 0.005). The rate of hospitalization revealed a direct relationship to the “level of concern”; ranging from 100% if major concerns, 42% if minor concerns, and 7% if no concerns raised, p = 0.001. Quality of life scores significantly worsened in patients with unintended hospitalizations at 6 (p = 0.046) and 12 months (p = 0.027) when compared to baseline scores. No significant differences in tremor scores between unintended and non-unintended hospitalizations were observed.

**Conclusions:**

The number and level of concerns raised during interdisciplinary Deep Brain Stimulation screenings were significantly related to unintended hospitalizations and to a reduced quality of life. The interdisciplinary evaluation may help to stratify risk for these complications. However, data should be interpreted with caution due to the limitations of our study. Further prospective comparative and larger studies are required to confirm our results.

## Introduction

Essential tremor (ET) is one of the most common movement disorders with a reported prevalence varying from 0.4% to 3.9% in population-based studies [1], and has an incidence which increases with age [2]. The core clinical symptoms of ET are postural and action/intentional tremor of the arms and head. Tremors of the voice, legs, or trunk are less common [3]. While ET was classically considered a monosymptomatic tremor disorder, non-motor clinical symptoms, including cognitive and mood issues, are now increasingly recognized as important features of the disease [4]. These symptoms frequently lead to a greater difficulty in performing activities of daily living (ADL) and this impacts negatively on quality of life (QOL) [5, 6].

Though tremors are successfully treated with medications in about 50% of patients for those with medically-refractory and disabling tremors, deep brain stimulation (DBS) of the ventralis intermedius nucleus (Vim) of the thalamus has been shown to be effective in reducing the shaking by 50% to 90% at long-term follow-up [7, 8].

Proper selection of surgical candidates is considered critical for achieving successful DBS outcomes. To date, there are no universally accepted criteria for assessing DBS candidacy, and selection has been based largely on the experience of each institution or alternatively on expert consensus [9]. At our institution, we perform a multi-specialty interdisciplinary preoperative assessment followed by a team discussion of risks, benefits, and candidacy. We suspect that many large DBS centers employ similar screening methods. Although these multi- or interdisciplinary DBS assessments have become an important topic and are widely accepted as an optimal model to select patients for surgical therapy, there exist little hard data supporting the use of a team in clinical practice.

In the present study, we aimed to examine whether detailed screenings by interdisciplinary DBS team members were associated with identification of post-surgical unintended hospitalizations (UH) and also with changes in QOL. We also aimed to examine the association between the levels of raised concerns from the interdisciplinary team with the rate of UH. We hypothesized that the screening process would identify patients at risk for post-operative complications and also those with a less robust QOL benefit.

## Materials and Methods

The present study was approved by the University of Florida (UF) Institutional Review Board (IRB). All patients signed an informed consent and data was stored in the UF INFORM database. Patient records were anonymized and de-identified prior to analysis. The UF INFORM system is a clinical-research database which provides information on a patient’s demographics, clinical, and functional characteristics.

### Study design and Settings

An observational cohort study of ET DBS patients was conducted at the UF Center for Movement Disorders and Neurorestoration from the period of January 2011 to February 2013.

### Patient Selection

Patients with a diagnosis of ET were identified from the INFORM database. Those who had received previous DBS surgery at an institution other than UF Gainesville, FL were excluded from the study. Inclusion criteria encompassed subjects diagnosed by a movement disorder trained-specialist according to internationally accepted criteria [10]. All subjects were referred to DBS therapy because of medically-intractable severe disabling tremors. Additionally, demographics and clinical scores using the Fahn-Tolosa-Marin Tremor Rating Scale (TRS) [11] were documented from each patient’s electronic medical record.

### UF DBS Interdisciplinary Team

All patients were pre-operatively and independently evaluated by seven team members including a movement disorders trained neurologist, a functional neurosurgeon, a neuropsychologist, a psychiatrist, a physical therapist, an occupational therapist, and a speech-language pathologist. Based on the final assessment from each screening specialty evaluation, patients were classified as having major, minor, or no concerns for future DBS therapy according to each specialist’s perspective. A concern was defined as a clinical finding on each evaluation which could place an individual patient at risk of surgical complications. The issues per specialty cited as contributing to the team’s level of concern are shown in Table 1. Definitions for levels of concern were: i) **Major Concern**: the evaluation raised concerns that the surgical risk possibly exceeded the benefit; ii) **Minor Concern**: the evaluation raised concerns that could potentially increase the risk of DBS, but the benefits were felt to outweigh the risks; **No Concern**: the interdisciplinary consensus did not express concerns for surgery. The risks and potential benefits for each DBS candidate as well as the proposed surgical interventions were discussed at an interdisciplinary team meeting. Consensus approval was reached prior to any intervention. Candidates not approved for DBS surgery by the interdisciplinary team were those deemed to have an unfavorable risk-benefit profile following evaluation and discussion. It was possible therefore to have one or more specialties express a major concern for DBS surgery and to still receive the operation.

**Table 1:** Issues cited as contributing to the team’s level of concern.

Specialty	Number of posible concerns	Major concern	Minor concern
Neurology	4	Accurate diagnosis	Age, Medical refractoriness of symptoms, Associated co-morbidities
Neurosurgery	3	-	Brain MRI findings (e.g. atrophy), Medications (e.g. anticoagulants, aspirin), Social history (e.g. smoker)
Neuropsychologist	2	Dementia	Cognitive impairment
Psychiatrist	3	Psychosis	Depression, Anxiety, Impulse control disorders
Physical therapist	2	Fall risk	Gait problems
Occupational therapist	1	-	Social work abilities
Speech-language pathologist	2	Dysphagia	Severity of dysarthria

### Unintended hospitalizations

Patients were queried at each DBS follow-up visit (monthly for the first 6 months and then at 1 year post-DBS) for the occurrence of any hospitalization. Hospitalization was then verified by medical record review documenting the underlying cause leading to the event. Admissions or hospital encounters for any medical reason were considered as an UH. Hospitalizations related to typical DBS care such as lead and neurostimulator (i.e. battery) implantations or regular battery changes were excluded.

### Changes in QOL

The changes in QOL were evaluated using the Medical Outcomes Study Short-Form Health Survey (SF-36) [12]. The SF-36 scale is a widely used general health outcome measure with four physical components (physical function, physical role, body pain, and general health) and four mental components (vitality, social function, emotional role and mental health). Each component ranges from 0 to 100, where higher scores indicate better QOL. All outcome measures for this study were obtained pre- as well as 6 and 12 months post-operatively.

### Statistical Analysis

Descriptive statistics (percentages, means and standard deviations) were used to describe demographics and clinical characteristics. Patient characteristics, clinical, and QOL assessments were compared between UH and non-UH using the Wilcoxon rank-sum test. Fisher exact test was used to assess UH by levels of concerns from pre-surgery assessments. The number of concerns and the UH were compared using the Cochran-Armitage analysis. The Jonckheere-Terpstra test was used to assess MOS-36 by levels of concerns from pre-surgery assessments. All calculations were performed using SAS software, version 9.1.3 (SAS Institute, Cary, NC, USA). Statistical significance was set at p < 0.05.

## Results

**Fig 1:**
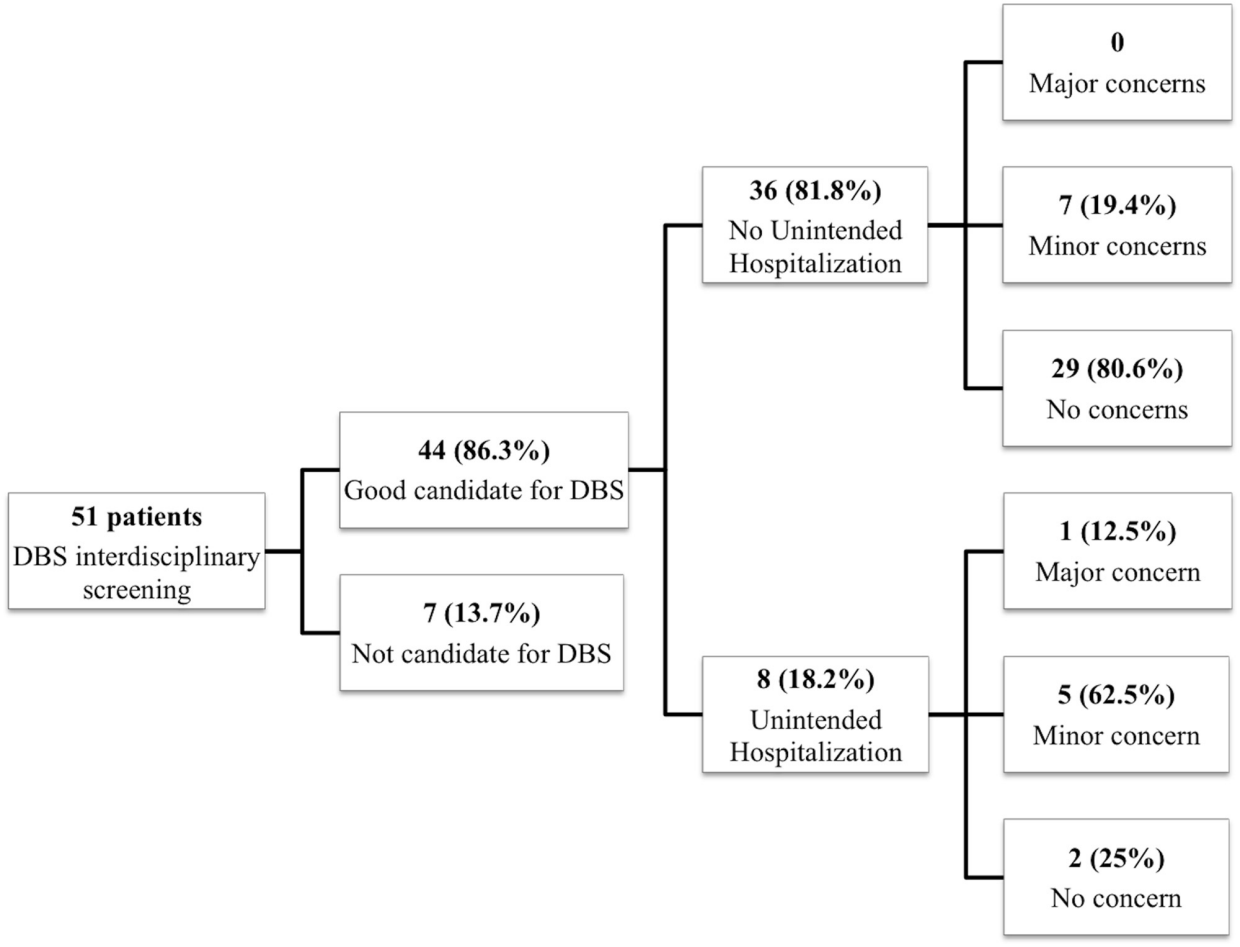
Number of patients per level of concern for those with and without UH.

Of the 51 ET cases assessed by the interdisciplinary DBS screening team, 44 (86.3%) were approved to receive DBS surgery. Male gender predominated (68%), with a mean age at surgery of 65.5 (SD = 10.3) years, and mean disease duration of 22.3 (SD = 13.5) years. Preoperative mean TRS scores were 53.7 (SD = 13.7) and mean SF-36 scores were 484.8 (SD = 154.9). Eight (18.2%) of the 44 cases experienced an UH following DBS. Fig 1 show the levels of concern for those with and without UH. Issues most commonly cited as contributing to reservations expressed by each service during screening were fall risk, age, comorbidities, cognitive, and swallowing concerns.

### Unintended hospitalizations

**Fig 2:**
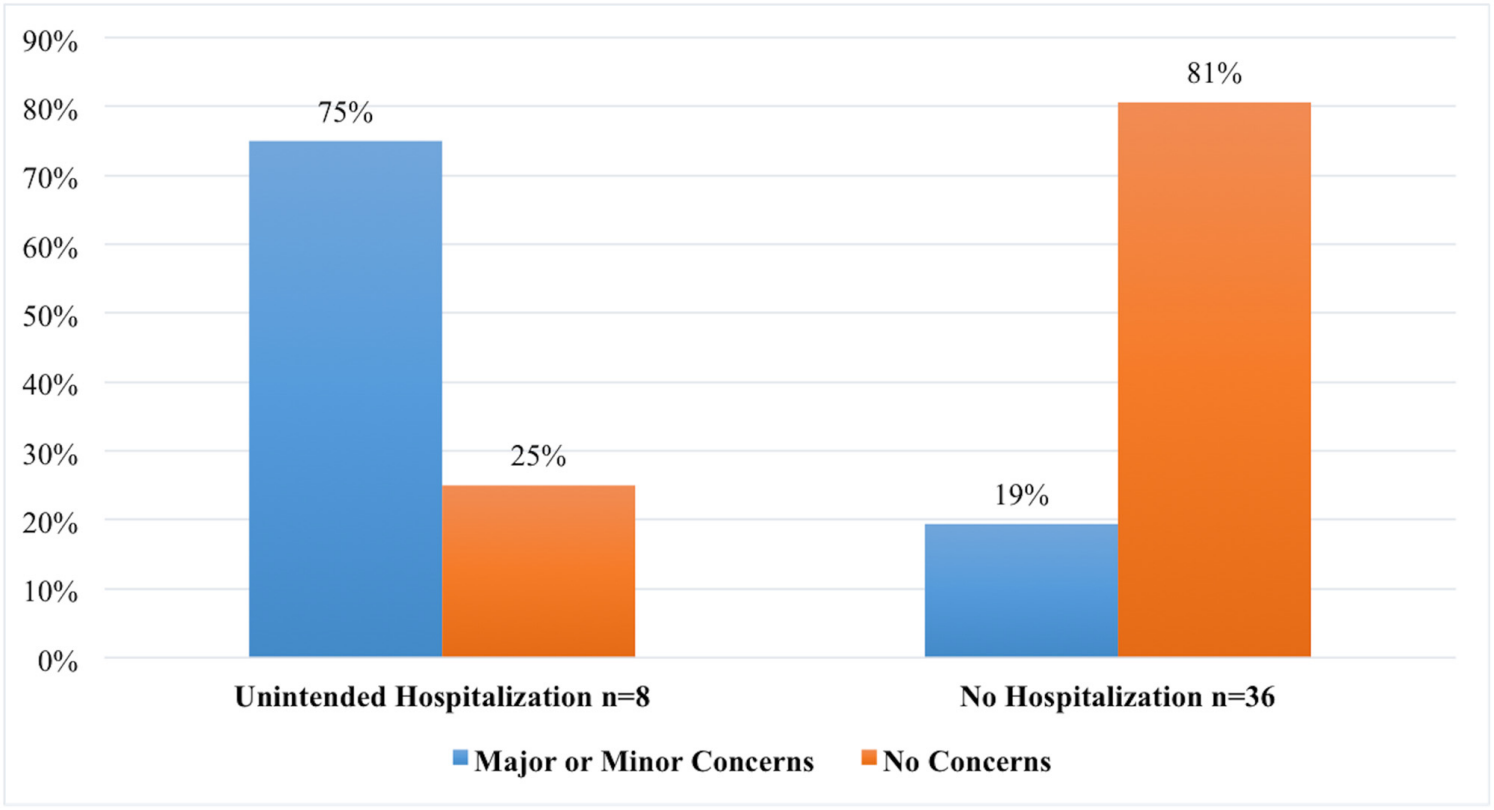
The relationship between unintended hospitalizations and level of concern. Patients with minor or major concerns experienced more frequently an unintended hospitalization when compared to those without concerns raised during the interdisciplinary meeting (p<0.005).

UHs were attributed to falls in 5 of our patients and the other UHs were deep venous thrombosis, an infection, and a venous infarction. Six of the 8 cases (75%) had either a major or minor concerns. The only one reported major concern in our cohort was a patient with dysphagia and severe dysarthria. In contrast, only 7 of 36 cases (19.4%) without an UH had concerns (p < 0.005), as shown in Fig 2. A comparison between the UH and non-UH groups is summarized in Table 2. No significant differences were observed regarding gender, age, disease duration, and baseline TRS and SF-36 scores. Patients with history of UH had significantly less improvement in SF-36 at 6 and 12 months post-surgery (p = 0.046 and p = 0.027, respectively). Although TRS scores were significantly better at 6 months in the non-UH group, this difference was not observed at 1 year follow-up.

**Table 2:** Demographic and clinical characteristics of patients.

		Patients (n = 44)		
	Overall	Unintended Hospitalization (n = 8)	Not Unintended Hospitalization (n = 36)	P value
Male:Female (%)	30:14 (68:32)	5: 3 (62.5: 37.5)	25: 11 (69.4: 30.6)	0.695
Age at surgery, years (SD)	65.5 ± 10.3	67.5 ± 8.6	65.0 ± 10.7	0.540
Disease duration, yrs (SD)	22.3 ± 13.5	22.5 ± 20.0	22.2 ± 12.0	0.971
Preoperative TRS	53.7 ± 13.7	53.6 ± 12.1	53.7 ± 14.2	0.927
Postoperative (6 months) TRS	31.3 ± 10.9	40.8 ± 8.8	29.0 ± 10.3	0.011*
Postoperative (12 months) TRS	30.1 ± 12.6	33.4 ± 11.4	29.3 ± 12.8	0.268
Preoperative SF-36, (SD)	484.8 ± 154.9	371.9 ± 171.6	509.9 ± 141.5	0.053
Postoperative (6 months) SF-36, (SD)	566.3 ± 166.0	439.0 ± 195.3	594.6 ± 147.3	0.046*
Postoperative (12 months) SF-36, (SD)	581.3 ± 154.6	434.4 ± 214.9	613.9 ± 118.7	0.027*

TRS; the Fahn- Tolosa- Marin Tremor Rating Scale, SF-36; the Medical Outcomes Study Short-Form Health Survey

The analysis further revealed a positive association between the number of concerns and frequency of an UH; the higher the number of concerns, the higher the frequency of UH (p = 0.002). Those patients who had 2 or 3 concerns had a 50% and 100% frequency of an UH, respectively (Table 3). Also, as the preoperative concern level classification shifted from “major” to “minor” to “no concern”, the frequency of UH decreased from 100% to 41.7% to 6.5%. These results have also been summarized in Table 4.

**Table 3:** Correlation between the number of raised concerns and unintended hospitalization.

		Unintended hospitalization	
Number of concerns	Total	Yes	No
3	2	2 (100.0%)	0
2	4	2 (50.0%)	2 (50.0%)
1	11	2 (18.2%)	9 (81.8%)
0	28	3 (10.7%)	25 (89.3%)

Cochran- Armitage analysis P = 0.002.

**Table 4:** Correlation between level of concerns and unintended hospitalization

	Total	Unintended hospitalization	
		Yes	No
Major concern	1	1 (100.0%)	0 (0.0%)
Minor concern	12	5 (41.7%)	7 (58.3%)
No concern	31	2 (6.5%)	29 (93.5%)

Cochran-Armitage analysis P = 0.001.

### Quality of life

The analysis revealed an inverse association between the levels of concern and the SF 36 scores, revealing that the higher the level of concern, the lower the QOL outcome score (p < 0.001 at 6 months, and p = 0.005 at 1 year post-DBS follow-up, respectively). These results have also been summarized in Table 5.

**Table 5:** Correlation between level of concerns and SF-36 total scores.

	Major concern n = 1	Minor concern n = 12	No concern n = 31	
Baseline	131	453.8 ± 142.8	508.3 ± 147.6	P = 0.121
6 months	180	460.8 ± 143.7	619.6 ± 139.9	P < 0.001
12 months	225	493.7 ± 166.9	626.6 ± 120.9	P = 0.005

## Discussion

A retrospective study was conducted to examine if data derived from pre-operative interdisciplinary screenings was helpful in understanding the one year risk of UH and to understand impacts on QOL. In total, our study revealed that 18% of the cohort had UH despite employing careful interdisciplinary screening. A major or minor concern was uncovered by the interdisciplinary DBS team in 75% of the UH group. In addition, the higher the number and the level of concern, the higher the frequency of UH. Moreover, the majority of subjects in the non-UH group had no concerns (80.6%). Although further studies are required to compare those DBS teams with and without interdisciplinary screening methods, our findings suggest that the use of interdisciplinary teams when screening for DBS surgery detect those patients at risk of postoperative UH. Though management of the concerns uncovered by an interdisciplinary team have not been previously adequately validated or addressed in the literature, an argument could be made that patients with concerns should undergo pre-operative counseling and closer post-operative follow-up care. These types of care issues were not addressed by our study. We can therefore only speculate that avoidance of UH likely will include factors such as fall prevention, drug optimization, and active monitoring of compliance with pharmacological and behavioral interventions.

In the UH group, falls were the most frequent reason for hospitalization, occuring in 5 of the 8 (62.5%) cases. Gait and balance issues have been reported in ET patients with and without DBS [13]. This finding is similar to a previous report where falling was also the most frequent reason for ET patients to visit the emergency room [14]. Though not definitive, this finding supports the potential importance of fall prevention therapy for ET patients with implanted DBS systems. Interestingly, the evaluation by PT did not always pick up the fall risk (only 3 of 8), highlighting the importance of the comprehensive screening process. It is important to also consider that post-operative falls could have been due to the characteristics of ET which include progressive gait and balance issues.

Another interesting finding derived from the current study was that the data from patients with a history of UH showed significantly less improvement in SF-36 at 6 and 12 months post-surgery (p = 0.046 and p = 0.027, respectively). In addition, the higher the level of concern, the lower the postoperative QOL. QOL in patients with ET may be affected by multiple reasons and may be especially impacted by tremor, social embarrassment, and ET associated associated mood changes [15, 16]. Previous studies have demonstrated the positive effects of ET DBS on QOL [17, 18]. Our study elucidated that QOL showed less improvement after DBS in the group who had UHs. What remains unknown is whether at risk groups for UH following ET DBS can be identified, and if an intervention can reduce the occurrence of this outcome.

Concerns expressed by 5 of the 7 interdisciplinary assessments were commonly associated with UH and these included neurosurgery, physical therapy, neurology, neuropsychology, speech, and swallowing therapy. Though the other disciplines (occupational therapy and psychiatry) were not vital contributors to UH and QOL, this should not be misinterpreted. These areas are likely important to the screening process and may be influencing different outcomes.

Most academic DBS centers utilize a limited interdisciplinary screening process including a neurologist, a neurosurgeon, and a neuropsychologist. Few use a psychiatrist, a physical and an occupational therapist. Given the prevalence of mood disorders in ET and the need for pre- and post-operative optimization, the psychiatrist can in select cases be an important contributor to the group. The prevalence of falling post-DBS would make a strong argument for the pre- and post-operative utilization of physical therapy. Finally, the occupational therapist can be an important team member especially in determining the pre- and post-operative activities of daily living needs. Additionally the occupational therapist has an important role in cases when a single unilateral lead is used, as the occupational therapy training uniquely positions this specialty to lead the dialogue to decide the most appropriate side for implantation.

This study was limited by the retrospective chart review methodology. This approach can miss or under-report complications. Additionally, the concerns documented in the study were determined based on interdisciplinary discussions among highly experienced DBS related medical practitioners, and may not be applicable to less experienced teams. Each team evaluated the candidates based on their own detailed quantitative assessment, though the ultimate discussion and concerns were expressed by each discipline in a simple qualitative manner. Further studies should be conducted to compare qualitative and quantitative screening methodologies, though we suspect both techniques will be needed to account for the complexity of the DBS screening process. Finally, the small size of our cohort and the absence of a control group without an interdisciplinary team evaluation, just by the classical team composed of neurology, neurosurgery, and psychiatry, limited the interpretation of the results. Further studies including outcomes from movement disorders centers screening for DBS candidates with the classical team composed of neurology, neurosurgery, and psychiatry, would be necessary to confirm our results.

## Conclusion

The current study demonstrated that the ET DBS interdisciplinary team approach provided important information on risk for UH. This study also showed that UH was associated with a worse QOL. The success of DBS therapy is known to be heavily dependent on the quality of candidate selection. Our study suggests that detailed screenings by interdisciplinary teams could be useful in assessing risk and predicting ET DBS outcome.
